# Medicine

**Published:** 1906-03

**Authors:** R. Shingleton Smith


### Progress of the Medical Sciences.

### MEDICINE.

**Graves's Disease.**—The treatment of this disease has occupied the attention of many workers and of many writers for some years past. It has been expected, and apparently with good reason, that this disease and myxœdema being the antithesis of each other, and thyroid treatment being so effective in the latter state, an anti-thyroidal treatment should be equally successful in the other. In spite of the numerous and repeated efforts of many pathologists and clinicians, it has to be admitted that treatment based on the theory that exophthalmic goitre is due to excessive or faulty thyroid function has not met with that success which was to be expected. A review of the recent work in this direction by Batty Shaw was quoted in the Long Fox Lecture (vol. xxiii., p. 337). The anti-thyroidin preparation of Merck, prepared by the method of Mœbius, is one of the best available preparations of the serum of thyroid-ectomised sheep or goats. Mœbius prefers the serum to any preparation of the milk, and one case is quoted by Batty Shaw[1] in which the daily dose, increased to 4.5 grammes, resulted in the disappearance of all symptoms except the proptosis. Such a dose becomes so expensive as to be absolutely prohibitive to all but wealthy patients, and, moreover, a very complete trial made by Hector Mackenzie[2] gave no good effects whatever. Accordingly, treatment by means of the milk, instead of the serum, of thyroidectomised goats has been instituted, and some interesting details of its results were given by Dr. Murray in his recent Bradshaw Lecture.[3] He considers that the preparation called Rodagen is worthy of more extended trial, but should

[1] *Organo-therapy*, 1905, p. 79. [2] *Brit. M. J.*, 1905, ii. 1081.

[3] *Ibid.*, ii. 1250.

be used with caution in doses not exceeding half a drachm. It is put up in tablet form, and sold by the Lanoline Company of Berlin at a price which is not unreasonable or prohibitive for use in special cases for short periods.

Dr. Murray also reports his own endeavours to produce a thyrolytic serum "which should exert a solvent action on the secreting cells of the thyroid gland, and so lead to their gradual and controllable solution and destruction without recourse to operative treatment."[1] Several cases are mentioned in which an anti-thyroid serum prepared from rabbits and goats was used, but Murray reports that no special effect could be traced to the use of this serum. Batty Shaw[2] also reports numerous attempts to produce a serum in one of the lower animals which would be capable of exerting a solvent effect upon the cells of the thyroid body, but he concludes his review with the remark "that experimental efforts to prove the possibility of developing a thyrolytic serum for therapeutic purposes have failed."

It is at least disappointing that there should be no better result than this for all the labour which has been expended on the inquiry. We must not, however, assume that the investigation has concluded, for the field is a hopeful one for continued observation and experiment, and it is not unlikely that success will one day crown the work. For the present we must admit that there is no certainty as regards the results of any form of gland treatment, or of serum treatment, in Graves's disease; accordingly we have to abide by the old lines based on the neuro-cardiac views of the pathology of the disease, and hence in the recent papers there is little advance on the methods of treatment laid down in the older text-books. We have learnt that all medicine is not limited to microbes and sera; the older neuro-cardiac theory still remains as the foundation of our treatment.

Many are the drugs which have been suggested and tried—so many in fact that the natural inference is that none of them has any uniform specific effect, but that all are found to be fickle and uncertain in their results. Accordingly, Mackenzie remarks[3] that drugs hold an altogether secondary place in the treatment of Graves's disease, and he emphasises the need for general hygienic methods. He mentions good food and an abundant supply of fresh air, and adds that "the open-air treatment is perhaps more easily carried out in the case of Graves's disease than in almost any other disease, because the patients are very tolerant of cold, always feeling better when the weather is cool, and they thoroughly enjoy being out in the fresh air. If you carry out open-air treatment in cases of Graves's disease I am sure you will be doing what is good for the patients." As regards change of air and climate, Mackenzie remarks that "the great thing is to get our patients to a place where they can get peaceful quiet and rest, with freedom from noise and

[1] Batty Shaw, *op. cit.* [2] *Op. cit.* [3] *Loc. cit.*

nuisances, where they can have real comfort and wholesome food, and where they can enjoy pure and bracing air among pleasant and cheerful surroundings." He does not advise sea-voyages or popular Swiss health resorts.

As regards food, it is obvious that in a disease where increased metabolism and wasting are so commonly present it is necessary to give an abundant, wholesome, and nourishing diet; hence the combination of good food and fresh air is just such as is commonly the routine practice in a sanatorium for consumptives. If weight is being lost, then an abundant supply of two to four pints of milk a day may be given, in addition to the ordinary diet.

Then again massage may sometimes be employed with advantage, but must be judiciously employed. A prolonged rest in bed is usually needful if the case be severe, but strict isolation is not commonly necessary. Murray observes[1] that the patient "should be induced to lead as quiet and easy an outdoor life as she can, freed as much as possible from anxiety, from undue mental effort, from uncongenial social duties, and from unsuitable physical exertion." So long as the patient is losing flesh the rest should be absolute, and everything must be done to encourage rest of mind as well as of body. Every disquieting influence must be avoided, and every encouragement towards recovery should be given.

Of the drugs from which much is expected, belladonna ranks as one of the first. The theory is that it diminishes the activity of the thyroid gland: this may be the case, but we have no proof that it is so. Murray has found it useful in some cases, and Mackenzie states that patients say they feel better when taking ten minims of the tincture three times a day. It seems to act best when the cardiac symptoms predominate, and it should be especially useful in those cases where excessive perspiration is a marked feature. Professor Mœbius states that he finds belladonna useless, and he wonders why it has maintained its popularity in spite of constant failure.[2]

Murray states that arsenic is one of the most useful drugs for routine treatment, small doses being given. A useful method is to order four or five minims of liquor arsenicalis to be taken

[1] *Loc. cit.*, p. 1248.

[2] The veteran Sir Samuel Wilks, Bart., in a private letter, writes as follows: "For some reason I was thought to be a sceptic in drugs, and so I wrote to say that I did not believe that for every ache and pain there was a drug to cure it . . . but there were medicines which alone and without any other aid had power to raise patients from their supposed dying beds. . . . some other drugs, and amongst them belladonna in Basedow's disease. Cases which were progressing to the bad I believed I absolutely cured by continuing for several months with some intermissions if specific symptoms of poisoning came on. I can't see how I could have been mistaken. I think there is little doubt that this drug has more immediate effect on the complaint than any other. Good doses for a few days produce a marked diminution of the more prominent symptoms."

three times a day, except during one week each month, or during the menstrual period, for six, eight, or even twelve months. When the pulse-rate is over 110, ten or fifteen minims of tinct. convallaria is added to the dose; and if nervousness be a marked feature, then ten grains of potassium bromide may be added for a few weeks at a time.

Other nerve sedatives in addition to the bromides are likely to be useful in a condition where so much nervous irritability exists. Caffein, antifebrin, phenacetin, or antikamnia are often useful.

It is curious to observe how much discrepancy exists in the various views with regard to digitalis and other heart sedatives. Murray states that convallaria commonly gives better results than either digitalis or strophanthus, but Mackenzie states that he has never seen the administration of digitalis or of strophanthus make any difference in the rate of the heart. This is surely not the usual experience, for in the later stages of the disease with actual heart incompetence digitalis acts with mathematical regularity, and in the milder cases the tachycardia is usually modified if not altogether controlled for a time. In the writer's experience digitalis for all purposes gives better and more reliable results than either strophanthus or convallaria, and in Graves's disease it rarely fails to give some relief, as it is the most useful method we have for controlling the too rapid action of the heart as well as counteracting the early, and more emphatically the later, stages of cardiac incompetence; no matter how advanced these may be in a case of Graves's disease, the patient may not be beyond the range of efficient treatment.

Sparteine has often been found to give good results, but its action is by no means comparable with that of digitalis in efficient doses.

Iodides have been so serviceable in the treatment of ordinary goitre that naturally they are often tried in cases of the goitrous tachycardia and exophthalmos, but the experience of Murray is quite the usual one, for he states that it not unfrequently aggravates the symptoms. This, he says, "is not surprising when we remember that thyroid secretion, which contains iodine in an organic combination, is being poured into the blood in excessive quantities," accordingly he considers that iodide of potassium is quite unsuitable and should not be given. On the other hand, Mackenzie states that "preparations of iodine are useful in cases where the goitre is large or where it is increasing in size. Lately I have been giving the syrup of hydriodic acid, which is better borne than iodide of potassium and seems as effective."

Phosphate of soda, in doses of 15 to 20 grains three times a day, has come into some favour, as also has chloride of calcium, but neither Murray nor Mackenzie can say anything better for them than that they will do no harm.

Local treatment of the goitre is commonly recommended. Iodine, so useful in cases of simple goitre, is externally of some use in Graves's disease; the red iodide of mercury ointment is also of some service. In very acute cases, or in acute exacerbation, the ice-bag applied to the goitre does something to reduce its size and to relieve the cardiac and gastric symptoms of the graver kind. In these cases it is sometimes needful to resort to morphia hypodermically and to feeding by rectum. Injections of iodine or of ergotine into the gland have often been tried, but they do not seem to be much in vogue at the present time. The latter drug is often given internally, but it has always seemed to the writer that it must be useless except when acting locally on the gland itself; formerly it was often used in this way by injection into one or other lobe of the gland, and sometimes with very good results, but it was a disagreeable method of treatment which few patients would continue for any length of time. Such preparations of iodine as Iodipin and Iothion should be very useful as local applications.[1]

Electricity has been much used—sometimes the galvanic current, sometimes Faradic has been preferred—but of late years both seem to have dropped into disuse, and the X-ray method has come into vogue. Electrical methods have been diligently used in the expectation that they would influence the cervical sympathetic as well as the thyroid, the eyes and the heart, but they have been for the most part disappointing. I am indebted to Dr. Kenneth Wills for the following notes on X-rays and the thyroid gland, from which it will be seen that further trials of X-ray treatment are most desirable, and may do more than gratify the patient's mind.

**X-rays and the Thyroid Gland.**—While treating a woman of 52 years for carcinomatous glands with the X-rays, Stegman[2] noticed that a large parenchymatous goitre, which was in the area rayed, decreased in size, losing one quarter of its bulk in six treatments; the clavicular glands were also found to have disappeared as far as palpation was concerned. He then tried a large, soft parenchymatous goitre in a girl aged 21, and found that after two exposures only the circumference of the neck had decreased from 39.8 centimetres to 36, and the dyspnœa was relieved.

Other observers have noticed a similar decrease in the size of parenchymatous goitres, but in many cases no obvious benefit

[1] Dr. Hawkins, of North Petherton, gives the following remarkable history: "An unmistakable case here some two years ago was so bad that I almost despaired as to the result. She was a married woman, whose child contracted scarlet fever: this was communicated to the mother when the Graves's disease was at its worst. The result was that the mother passed through a normal scarlet fever attack, made a good recovery, and the symptoms of her Graves's disease quite disappeared. She is now fat and well, although the goitre is as marked as ever."

[2] *Med. Electrol. and Radiol.*, Aug., 1905.

was observed. However, some good results have been obtained by Görl[1] and Carl Beck.[2]

The former has had "encouraging" results in seven cases. He considers that the action of the rays is upon the parenchyma of the gland itself, and not upon the blood vessels, which are commonly believed to be immediately affected. He finds that these patients have an unusually sensitive reaction to the X-rays.

Carl Beck has practised unilateral extirpation of the thyroid gland, and has followed it up with irradiation to the remainder of the gland. In two cases the results have been gratifying: the tachycardia and the dyspnœa which remained after the operation and the exophthalmos disappeared, also the general health improved. In one of the cases the patient was described as "perfectly cured" in a few months after the operation.

Robert Abbé[3] has had good results in a case of exophthalmic goitre by means of a radium tube embedded in the tumour of the thyroid. Miss A. B., age 21, with enlargement of thyroid and thyroidism, had such great difficulty in breathing on exertion that she was incapacitated from ordinary household duties. On July 28th a small incision, under cocaine, was made in median line, isthmus of thyroid exposed, and a sterilised tube of radium was buried in the enlarged median lobe an inch deep. There were 10 centigrammes of Carec radium (300,000 radio-activity). This was kept in place for twenty-four hours. Improvement was not immediate, but four months subsequently, although there was some tachycardia (105–120 pulse rate), the patient felt well, could play tennis, had lost headache, tremor and ocular protrusion. The thyroid had decreased to one-sixth of its original size in every diameter.

R. Shingleton Smith.

1 *München. Med. Wchnschr.*, May 16th, 1905; *Med. Electrol. and Radiol.*, July, 1905.

2 *Berl. Klin. Wchnschr.*, May 15th, 1905. 3 *Archi. Röntg. Ray*, March, 1905.

